# Development of molecular tools for diagnosis of Alzheimer’s disease that are based on detection of amyloidogenic proteins

**DOI:** 10.1080/19336896.2021.1917289

**Published:** 2021-04-29

**Authors:** Konstantin Y. Kulichikhin, Sergei A. Fedotov, Maria S. Rubel, Natalia M. Zalutskaya, Anastasia E. Zobnina, Oksana A. Malikova, Nikolay G. Neznanov, Yury O. Chernoff, Aleksandr A. Rubel

**Affiliations:** a Laboratory of Amyloid Biology, St. Petersburg State University, St. Petersburg, Russia; b I.P^.^ Pavlov Institute of Physiology, Russian Academy of Sciences, St. Petersburg, Russia; c SCAMT Institute, ITMO University, St. Petersburg, Russia; d V.M. Bekhterev National Research Medical Center for Psychiatry and Neurology, St. Petersburg, Russia; e Sirius University of Science and Technology, Sochi, Russia; f School of Biological Sciences, Georgia Institute of Technology, Atlanta, GA, USA; g Department of Genetics and Biotechnology, St. Petersburg State University, St. Petersburg, Russia

**Keywords:** Alzheimer’s disease, amyloid beta, Aβ oligomers, blood, early diagnostics

## Abstract

Alzheimer’s disease (AD) is the most common form of dementia that usually occurs among older people. AD results from neuronal degeneration that leads to the cognitive impairment and death. AD is incurable, typically develops over the course of many years and is accompanied by a loss of functional autonomy, making a patient completely dependent on family members and/or healthcare workers. Critical features of AD are pathological polymerization of Aβ peptide and microtubule-associated protein tau, accompanied by alterations of their conformations and resulting in accumulation of cross-β fibrils (amyloids) in human brains. AD apparently progresses asymptomatically for years or even decades before the appearance of symptoms. Therefore, development of the early AD diagnosis at a pre-symptomatic stage is essential for potential therapies. This review is focused on current and potential molecular tools (including non-invasive methods) that are based on detection of amyloidogenic proteins and can be applicable to early diagnosis of AD.

**Abbreviations**: Aβ – amyloid-β peptide; AβO – amyloid-β oligomers; AD – Alzheimer’s disease; ADRDA – Alzheimer’s Disease and Related Disorders Association; APH1 - anterior pharynx defective 1; APP – amyloid precursor protein; BACE1 – β-site APP-cleaving enzyme 1; BBB – brain blood barrier; CJD - Creutzfeldt-Jakob disease; CRM – certified reference material; CSF – cerebrospinal fluid; ELISA – enzyme-linked immunosorbent assay; FGD – ^18^F-fluorodesoxyglucose (2-deoxy-2-[^18^F]fluoro-D-glucose); IP-MS – immunoprecipitation-mass spectrometry assay; MCI – mild cognitive impairment; MDS – multimer detection system; MRI – magnetic resonance imaging; NIA-AA – National Institute on Ageing and Alzheimer’s Association; NINCDS – National Institute of Neurological and Communicative Disorders and Stroke; PEN2 – presenilin enhancer 2; PET – positron emission tomography; PiB – Pittsburgh Compound B; PiB-SUVR - PIB standardized uptake value ratio; PMCA – Protein Misfolding Cycling Amplification; PrP – Prion Protein; P-tau – hyperphosphorylated tau protein; RMP – reference measurement procedure; RT-QuIC - real-time quaking-induced conversion; SiMoA – single-molecule array; ThT – thioflavin T; TSEs – Transmissible Spongiform Encephslopathies; T-tau – total tau protein

## Introduction

Alzheimer’s disease (AD) is a fatal neurodegenerative disorder and the most common form of dementia in late life, accounting for an estimated 60% to 80% of cases [1]. Most cases of AD are sporadic, with only a small fraction (less than 5%) being heritable [2]. The majority of sporadic AD patients are older than 65, and after this timepoint, the risk of AD development is doubled every five years, reaching more than 30% by the age of 85 [3]. From a histological point of view, AD is characterized by the appearance of proteinaceous deposits in the brain: extracellular plaques that are formed by amyloid β (Aβ) peptide, and intracellular neurofibrillary tangles, formed by tau protein. Aβ plaques and neurofibrillary tangles can be stained with amyloid-specific dyes such as Congo Red or Thioflavin S [4,5]. These amyloid deposits are a distinctive feature of AD differentiating it from other disorders that can lead to dementia [6,7]. Notably, deposition of Aβ usually begins first, followed by appearance of tau tangles, and then by cognitive impairment [8].

Previously, AD had been diagnosed by the application of several clinical criteria standardized in 1984 by the National Institute of Neurological and Communicative Disorders and Stroke (NINCDS) and the Alzheimer’s Disease and Related Disorders Association (ADRDA) workgroup based on the analysis of patient’s medical history; results of neurological, psychiatric and clinical examinations, neuropsychological tests and laboratory studies [9]. These criteria did not take into account specific biochemical features of AD, and thus they could establish diagnosis only as *probable AD* or *possible AD*. Diagnosis of *definite AD* could be made only when *probable AD* is additionally confirmed by histopathological examination of biopsy or autopsy material [9].

These criteria were revised in 2011 by the National Institute on Ageing and Alzheimer’s Association (NIA-AA) workgroup to incorporate scientific knowledge accumulated to date [10]. Based on neuroimaging techniques and cerebrospinal fluid (CSF) analysis, AD pathology biomarkers, such as Aβ deposition in the brain, Aβ42 and pathologic tau level in CSF, have been added to clinical criteria. The observation that AD pathological events (neurofibrillary tangles) can be present in the absence of any clinical symptoms [11] has also been taken into account building a basis for distinguishing between preclinical and clinical stages of AD. The course of the disease has been divided into three stages on the basis of both degree of cognitive impairment and AD biomarker profiles [10], namely: preclinical stage, characterized by normal cognitive ability; prodromal stage, characterized by mild cognitive impairment; and the dementia stage, characterized by functional impairment. The separate diagnostic recommendations have been formulated for each stage [12–14]. The clinical criteria of cognitive functions are still a basis for diagnosis, whereas biomarker information provides additional supportive evidence and is proposed to be used in clinical research and trials [10].

The NIA-AA has further updated the AD definition [6,7] for observational and interventional research, but not routine clinical care. According to the new definition, biomarkers dynamics reflects the progression of key pathological elements of AD. These markers have been separated into three groups: 1) **A**β deposition; 2) accumulation of pathogenic **T**au, and 3) **N**eurodegeneration, termed altogether as **AT**(**N**) system. **A** and **T** biomarkers reflect neuropathological changes specific to AD, whereas **(N)** can have a background other than AD and thus placed in parentheses.

Use of biomarkers allows to define the preclinical stage AD, characterized by the appearance of AD molecular characteristics without signs of cognitive impairment [12]. The full course of detectable AD may encompass from 15 to 25 years, with an asymptomatic period taking from 7.6 to 13 years depending on age, sex and presence of genetic predisposition, for example, *APOE4* allele [15]. Early identification of AD pathology at the preclinical stage could allow therapeutic intervention when such a therapy becomes available. Several pharmaceutical companies are now working on drugs for treating AD [16,17].

According to the Alzheimer’s Association Annual Report 2020, 5.8 million people 65 and older lived with AD dementia in the US in 2020, and this number is expected to reach 13.8 million by 2050 [1]. In 2020, AD and other dementias cost the US 305 USD billion, and these expenses are expected to rise to 1.1 USD trillion by 2050 [1]. It has been estimated that a hypothetical disease-modifying intervention that delayed the AD onset for five years would reduce the number of AD patients by 41% and costs of AD in the US by 40% by 2050 [18]. Thus, the development and implementation of biomarker-based methods for early diagnostics of AD at the preclinical, asymptomatic stage are of great social and economic importance.

## Current molecular biomarkers of AD: aggregate imaging and CSF analysis

Amyloid plaques and tangles are specific features that define AD as a unique neurodegenerative disease among disorders that cause dementia [7]. This drives most methods and approaches currently used for biochemical diagnostics of AD.

### Detection of Aβ

Amyloid plaques are composed of 4 kDa peptide [19], which is known today as amyloid β, or Aβ. Aβ isoform that prevails in plaques consists of 42 amino acid residues and is termed Aβ42. This peptide is a short fragment of amyloid precursor protein (APP) that is cleaved out by specific proteases, termed β- and γ-secretases. APP is a transmembrane protein with a single transmembrane domain. It is represented in humans by several polypeptides – APP695, APP751 and APP770, containing 695, 751 and 770 amino acid residues (a.a.), correspondingly. The diversity of APP isoforms originates from alternative splicing of APP gene and subsequent post-translational modifications [20,21]. APP751 and APP770 are present in all tissues, whereas APP695 is the most abundant form of the neurons. β-secretase (Beta-site APP-Cleaving Enzyme 1 – BACE1) is a monomeric protein that cleaves the APP cytosolic domain. γ-secretase is a multimeric complex of four proteins (presenilin, nicastrin, presenilin enhancer 2 – PEN2 and anterior pharynx defective 1 – APH1), and its cleavage site is embedded into membrane [22]. γ-secretase can cleave APP at several sites and produce various isoforms of Aβ: Aβ38, Aβ39, Aβ40 and Aβ42. Alternatively to γ-secretase, APP can be cleaved by α-secretase in the middle of Aβ sequence and thus be withdrawn from the amyloid-generation pathway.

Aβ42 is present in CSF [23] and its level is significantly decreased in CSF of AD patients [24], apparently due to ‘trapping’ of Aβ42 in brain. Association between the decrease of Aβ42 in CSF and accumulation of amyloid plaques in AD patients brains has indeed been reported [25]. Implementation of the positron emission tomography (PET) using ^11^C-labelled thioflavin-S analogue (Pittsburgh Compound B – PiB) opened an opportunity for the pre-mortem monitoring of fibrillary Aβ deposits in brain [26]. Elderly people who had lowered level of Aβ42 in CSF were also PET-positive and *vice versa*, irrespectively of whether they had AD symptoms or were cognitively unimpaired [27]. Results of further studies indicate that the decrease of Aβ42 levels in CSF precedes plaques formation in the brain [28].

The performance of the Aβ42 level in CSF as an AD biomarker may be substantially improved when it is presented as a ratio to the CSF level of Aβ40 [29]. Aβ40 is the most abundant proteolytic fragment of APP, whose concentration does not significantly change in the course of AD. Normalization of Aβ42 levels by Aβ40 levels helps to neutralize variations in the amounts of total Aβ [30] and impacts of natural CSF dynamics and/or pre-analytical manipulations that have a similar effect on Aβ42 and Aβ40 [31]. Aβ42/Aβ40 ratios show better correspondence to the PET-positive status of patients compared to Aβ42 levels per se. Therefore, ratio of Aβ42 to Aβ40 in CSF is recommended as the most prominent value for the improvement of preclinical AD diagnosis [32].

### Detection of tau

AD associated neurofibrillary tangles are composed of hyperphosphorylated tau protein [33], making it a useful candidate for an AD biomarker. Normally, Tau is a microtubule-associated protein located in the neuronal axons (see [34] for review). Due to alternative splicing, six different isoforms of tau, varying from 352 to 441 amino acids residues in length and from 50 to 65 kDa in molecular weight, are found in the cells. Hyperphosphorylation is proposed to disrupt the ability of tau to bind and stabilize microtubules in neurons, leading to disassembly of microtubules, defects in axoplasmic flow and loss of neuronal connectivity. An increase in total levels of tau protein (T-tau) was detected in CSF of AD patients (see [35] for review), however a similar increase was reported for acute neuronal damage or other neurodegenerative disorders such as Creutzfeldt-Jakob disease, CJD [36]. On the other hand, an increase in phosphorylated tau (P-tau) is specific to AD and helps to discriminate it from other neurodegenerative processes. Marked increase in P-tau phosphorylated at middle domain (Thr-181, Ser-199, Thr-231, Thr-235) and at C-terminal domain (Ser-396, and Ser-404) sites was detected in CSF of AD patients (see [31], and references therein). In contrast, P-tau levels in CSF are not increased in CJD, where massive brain degeneration is not accompanied by tangles appearance [36], or after acute brain damage, or ischaemic stroke [37]. Recently, the method of pre-mortem observation of tangles using PET with ^18^F-flortaucipir as the tau-amyloid-specific dye has been developed [38]. It has been found that preclinical AD patients showed normal tau PET imaging despite elevated levels of both P-tau and T-tau in CSF [39]. However, ligand binding was correlated with the degree of brain atrophy measured by MRI and severity of cognitive impairment in prodromal and demented AD patients [39].

Overall, existing data show that the increase in total tau in CSF reflects degeneration in CNS without identifying the exact reason for this process, whereas simultaneous increase in P-tau levels specifically points to AD.

### Value of CSF- and imaging-based diagnostics

To sum up, Aβ plaques detected by PET, and levels of Aβ42 (especially when normalized by levels of Aβ40) and tau (specifically, P-tau) in CSF serve as core biomarkers for presymptomatic detection of AD, and are in the process of implementation into clinical practice for early AD diagnosis. Several immunoassay kits for Aβ42, Ab40, T-tau, and P-tau detection in CSF are already commercially available, although primarily for research use.

A mass spectrometry-based reference measurement procedure (RMP) for detection of Aβ42 levels in CSF was developed recently, and certified reference materials (CRMs) were produced (see [31] and references therein). These CRMs can be used for calibration of assay results and making the data obtained by different laboratories compatible [31]. Fully automated systems for measuring core AD biomarkers are produced by several companies. For example, Roche’s Cobas Elecsys system allows for the simultaneous measurement of Aβ42, T-tau, and P-tau levels in a CSF sample in just 18 min [40].

**Figure 1.**
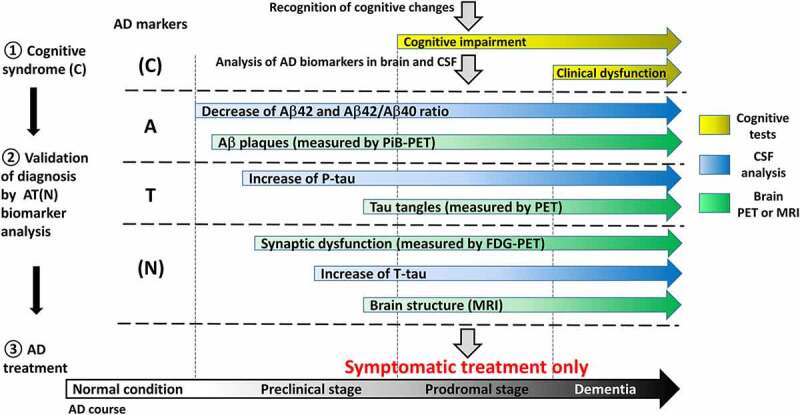
**Current state of AD diagnostics using biomarkers**. AD biomarkers are grouped and marked as it is proposed by Jack et al. [6] as reflecting different sides of AD pathological process. **A** – biomarkers related to Aβ pathology (CSF Aβ42, CSF Aβ42/Aβ40 ratio and Aβ plaques measured by PiB-PET); **T** – biomarkers related to tau pathology (CSF P-tau and tau tangles measured my PET); (n) – biomarkers of neurodegeneration (synaptic dysfunction measured by FGD-PET, CSF T-tau and brain structure analysed by MRI); (c) – cognitive symptoms (mild cognitive impairment at prodromal stage and clinical dysfunction at dementia). Similar to **(N), (C)** can have a background other than AD and thus placed in parentheses. Despite cognitive syndromes are the consequences of neurodegeneration, **(C)** is placed on the top of this scheme as nowadays AD diagnostics begins from the recognition of cognitive changes, when the patient is suspected to be already in the prodromal stage of the disease. Further analysis of core AD biomarkers from **A** and **T** blocks confirms or rejects the diagnosis, whereas **(N)** biomarkers level help to estimate the degree of degenerative processes in CNS

Despite recent progress in identification of AD core biomarkers and their potential applicability to a presymptomatic stage, in reality these biomarkers are typically applied mostly to patients, already exhibiting the symptoms of cognitive impairment or dementia (Figure 1). Preventive check-up to reveal AD at preclinical stage is irrelevant today for several reasons. The first reason is that both PET and lumbar puncture (used to take CSF samples) are invasive procedures, associated with uncomfortable feelings and even health risk for a patient. At second, lumbar puncture and analysis of four AD biomarkers in CSF may cost up to 200 euro, whereas a PiB-PET is 10–15 times more expensive [32]. Finally, no adequate therapy can be applied today to cure or to slow down the course of AD. It should also be noted that while changes in Aβ42, T-tau, and P-tau levels in CSF appear well before cognitive symptoms, by their nature they reflect neurodegenerative processes that have already progressed to a significant extent. Therefore, neither CSF analysis nor PET is suitable for detection of preclinical AD during routine examination of healthy people, and even these ‘early’ detection techniques might come too late for potential therapeutic interventions. Thus, it is not clear to which extent these procedures can be applied to testing effectiveness of prospective disease-modifying drugs that might become available in near future (see [16,17]).

### Other potential biomarkers of AD

In addition to amyloid-β and tau protein, which are core biomarkers of AD, various other compounds, such as other proteins, phospho- and sphingolipids, amino acids and non-coding RNAs have been identified as candidates for AD biomarkers (for review, see [41–43]). While these approaches are useful for clinical purposes, most of them reflect pathological consequences of already developed AD, rather than molecular events leading to pathology, and therefore, cannot be used for early presymptomatic diagnostics. For metabolic features that can possibly identify conditions promoting initial AD stages, it is usually not clear if they are specific to AD or may also apply to other dementias.

According to amyloid cascade hypothesis, the deposition of the Aβ peptide and tau protein in the brain are central events in AD pathology [44], and this concept is accepted by NIA and AA in their definition of AD [3,4] mentioned above. Changes of Aβ42 level, Aβ42/Aβ40 ratio, P-tau level in CSF and the appearance of Aβ42 plaques in the brain are the earliest events of AD and take place long before appearance of cognitive changes [8]. If polymerization of Aβ and tau represents early triggering events in AD, detection of these events at the molecular level provides the best hope for early diagnostics. Recent failures of some Aβ-targeting therapies [45] do not contradict this notion, because: a) these therapies typically did not address Aβ polymerization specifically, and b) they were applied at late disease stages, when neuronal damage becomes irreversible. Thus, we will provide a detailed consideration of only such new molecular tools for AD diagnostics that are based on monitoring of Aβ and tau proteins, and can therefore be potentially applicable to early pre-symptomatic stages of AD. It has to be noted that, typically, these approaches have been developed and validated in academic laboratories on limited sets of samples, but are not yet implemented into clinical practice and need further clinical validation.

## Novel approaches to development of molecular diagnostic tools for AD

### Comparison of CSF- and blood-based assays

Blood is strongly preferable to CSF in regard to accessibility, sampling techniques, applicability of potential diagnostic tools to routine non-invasive testing, and longitudinal clinical evaluations with repeated sampling [31]. However, detection of AD biomarkers in blood is associated with a variety of complications. As CSF is the nearest to the brain parenchyma, proteins secreted from brain’s extracellular space are directly accessible by lumbar puncture. In contrast, brain proteins have to pass the blood-brain barrier (BBB) for entering blood. BBB acts as a selective filter, so that only a small fraction of brain proteins enters the bloodstream. These brain proteins may be cleaved, otherwise modified or degraded before or after passing BBB. Finally, blood plasma contains a variety of background proteins, some of them present at high levels, and a relatively tiny amount of brain proteins has to be detected within this complex mixture. This is why additional validation and adaptation of sample preparation and measurement procedures are required when detection assays used for CSF are adopted for blood samples. For example, concentration of Aβ is approximately 50 times lower in blood plasma compared to CSF [46], whereas levels of background proteins are about 100-fold higher in blood plasma [47]. Generalized results of 22 studies conducted between 1999 and 2014 (see [35] for review) failed to detect any significant differences in Aβ levels (mostly measured by ELISA) between AD patients and control groups. This indicates that either modifications of existing assays, in order to increase their sensitivity, or development of novel analytical techniques is necessary for identifying AD biomarkers in blood. Some approaches to solving this problem are considered in the following sections.

### Single molecule array – SiMoA

**Figure 2.**
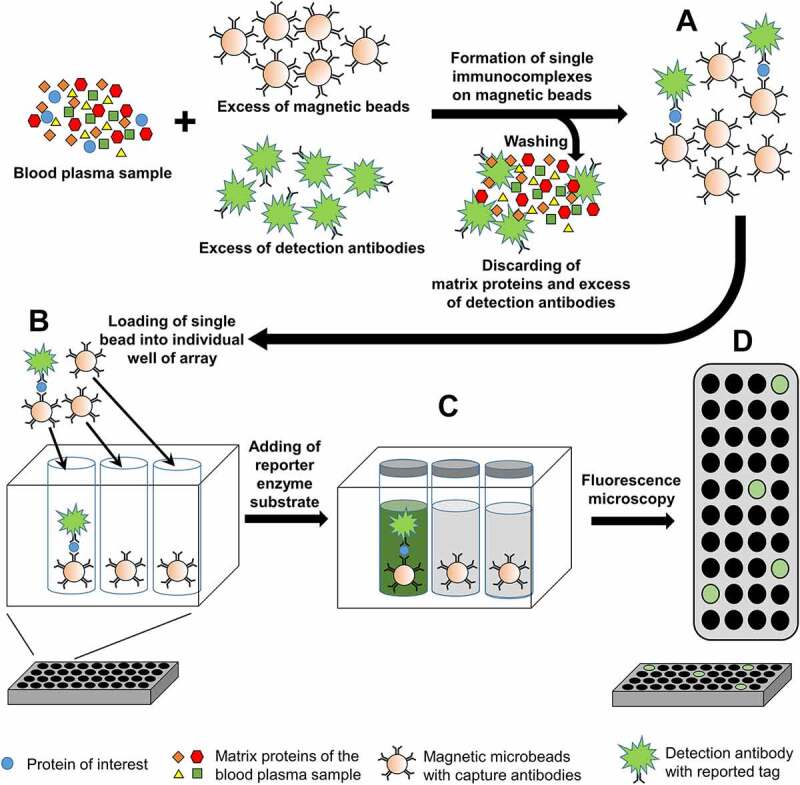
**The principle of Single Molecule Array (SiMoA). A** – Blood plasma sample is mixed with the excess of magnetic microbeads (2.7 μm diameter) with capture antibodies and detection antibodies. The number of beads added to the blood plasma sample exceed the number of molecules of protein-of-interest and thus each single bead will carry either single or no immunocomplex; **B** – Single beads with or without immunocomplex are loaded into 50 femtoliter well (4.5 μm diameter, 3.25 μm depths) of array. Rissin et al. [48] used 2-mm-wide array possessing 50,000 wells; **C** – Reporter enzyme substrate is added and the well is sealed with silicon gasket. Activity of single molecule of reporter enzyme in 50 fl volume is enough to generate fluorescence signal detectable by fluorescence microscopy; **D** – Fluorescence image of a section of the array after the signals from single molecules of reporter enzyme are generated

One approach allowing detection of serum proteins in blood plasma at subfemtomolar (below 10^−15^M) concentrations is single-molecule enzyme-linked immunosorbent assay, or a **Si**ngle-**Mo**lecule **A**rray, SiMoA [48]. In this technique (Figure 2), protein of interest is captured from the bulk solution on microscopic beads (2.7 μm diameter) decorated with specific antibodies and then the immunocomplexes (one or zero labelled target protein molecules per bead) are labelled with an enzymatic reporter capable of generating a fluorescent product. After isolating the beads in 50-fl reaction chambers designed to hold only a single bead, fluorescence imaging is applied to detect single protein molecules. The concentration of protein in bulk solution is correlated to the percentage of chambers that carry a bead with protein molecule trapped and thus capable of generating fluorescence signal [48].

The SiMoA protocol has been adopted for Aβ quantification [49] and used to measure levels of Aβ42 and Aβ40 in blood plasma samples of individuals with AD-derived dementia, with mild cognitive impairment (MCI, possibly including early stages of AD), and without signs of cognitive impairment [50]. A weak positive correlation between levels of both Aβ42 and Aβ40 in plasma and CSF, as well as a negative correlation between Aβ42 levels in plasma and brain amyloid deposition measured with PET have been reported. Nevertheless, in AD patients at preclinical or prodromal AD stages plasma concentration of Aβ42 was just moderately decreased whereas Aβ40 levels were unchanged. In AD patients with dementia symptoms, levels of both Aβ42 and Aβ40 in plasma were reduced. Both observations were in contrast to the noticeable decrease in Aβ42, but not in Aβ40 levels in CSF already at preclinical stage of AD (as explained above). Additionally, elevated level of Aβ in blood plasma but not in CSF has been found to be associated with other pathologies, such as hypertension, diabetes and ischaemic heart disease [50]. This indicates that Aβ metabolism in the periphery may be prominently different in comparison to the brain and thus Aβ markers measured in blood plasma did not show diagnostic value in case of AD [50].

### Immunoprecipitation followed by mass spectrometry assay

**Figure 3.**
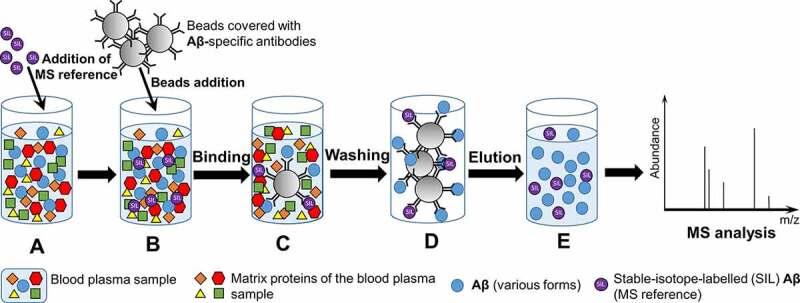
**Principle of the ImmunoPrecipitation followed by Mass Spectrometry (IP-MS) technique. A** – Stable isotope labelled (SIL) Aβ (MS reference) is added to the sample of blood plasma; **B** – Beads covered with Aβ-specific antibodies are added to the sample. **C** – Aβ and SIL-amyloid-β are bind by the beads. **D** – Beads are washed to remove matrix protein of blood plasma sample. **E** – Elution of Aβ and SIL-amyloid-β from the beads for further MS analysis

Another approach applied to the analysis of Aβ40 and Aβ42 in blood is based on immunoprecipitation (IP), followed by mass-spectrometry (MS) [51] (Figure 3). IP enriched the sample by proteins of interest, while MS technique, using stable-isotope-labelled Aβ peptide as a reference, allowed a precise determination of protein quantity. Nakamura et al. [52] improved the IP-MS protocol by incorporating a second round of IP, and found that Aβ40/Aβ42 ratio in blood plasma is significantly lower in individuals with brain Aβ-positive status, as determined by PET using PiB or other ligands. In addition, ratio of plasma APP_669-711_ (another proteolytic fragment of APP, see Figure 1) to Aβ42 was used and demonstrated even better correlation with Aβ deposition in brain than Aβ40/Aβ42 ratio. Both metrics are also correlated with Aβ42 level in CSF, reflecting concordant dynamics of these AD biomarkers in CSF and blood. Schindler et al. [53] confirmed the correspondence between plasma Aβ42/Aβ40 ratio and amyloid PET status, as well as P-tau181/Aβ42 ratio in CSF by using the same IP-MS approach, and pointed to the existence of the group of PET-negative cognitively normal individuals with decreased Aβ42/Aβ40 ratio in plasma; they proposed that such individuals may represent early stages of AD development that precede plaque formation. Based on mathematical modelling, such individuals have a 15-fold higher risk of development PET-positive amyloid assemblies in brain than individuals with a normal plasma Aβ42/Aβ40 ratio during the next six years [53]. Further studies are needed to confirm or reject this hypothesis.

The IP-MS-based approach has also been applied for precise detection of hyperphosphorylated tau isolated from brain and CSF. Twenty-nine distinct phosphorylated sites have been identified by MS in a full-length tau, isolated from soluble fraction of the human brain lysates, and 12 sites were identified in a truncated tau isolated from CSF [54]. Further analysis discriminated between several AD-specific P-tau isoforms, of which two, P-tau217 and P-tau205, appear to be of most interest. Level of P-tau217 was increased at fivefold in CSF of AD patients, as compared to the control group, whereas level of P-tau181, a more routinely used marker, was increased at only 1.3-fold [54]. P-tau217 had better performance than P-tau181 in discriminating AD patients from those with other neurodegenerative diseases and control subjects [55]. Levels of both P-tau181 and P-tau217 begin to rise in the early stages of AD, approximately 20 years before the appearance of detectable tau aggregation in brains [56]. On the other hand, levels of P-tau205 were increased in CSF of patients with later stages of AD, and correlated with elevated T-tau level and brain atrophy [56]. The IP-MS-based technique was also applied to demonstrate that levels of P-tau181 and P-tau217 in blood plasma and CSF correlate [57]. Importantly, measurement of P-tau217 levels in either blood plasma or CSF allows to distinguish Aβ amyloid-positive (but tau PET-negative) participants from controls. This suggests that changes in P-tau levels in body fluids occur before accumulation of tau aggregates at detectable levels in brain, and may therefore reflect abnormal tau metabolism occurring concomitantly with appearance of Aβ aggregation [57].

## Techniques based on amplification of misfolded protein conformations

Low concentrations of AD core biomarkers in blood plasma, technical complexity of procedures, such as SiMoA and MS that are used for their detection, as well as the need for costly equipment that is required to run such assays are underlining the necessity for searching of new biomarker(s) that are directly associated with AD pathology and for novel approaches of their detection in body fluids that would be able to amplify an initially weak signal.

Detection of Aβ oligomers (AβO), that appear early in the pre-symptomatic stage of disease and are directly associated with AD pathogenesis [58,59], can potentially be adjusted for pre-symptomatic diagnostic purposes. However, concentrations of AβO in blood plasma are in the range similar to those of Aβ42 [60], thus direct measurement of AβO contents is impractical. However, as in the case of other amyloidogenic proteins, Aβ oligomers can specifically seed polymerization of the soluble Aβ peptide, which is one of the major hallmarks of AD and is responsible for the spread of Aβ aggregation in AD brain (see [61] for review). This property allows amplification of Aβ oligomers via conversion of the artificially produced monomeric Aβ substrate into the polymeric form.

**Figure 4.**
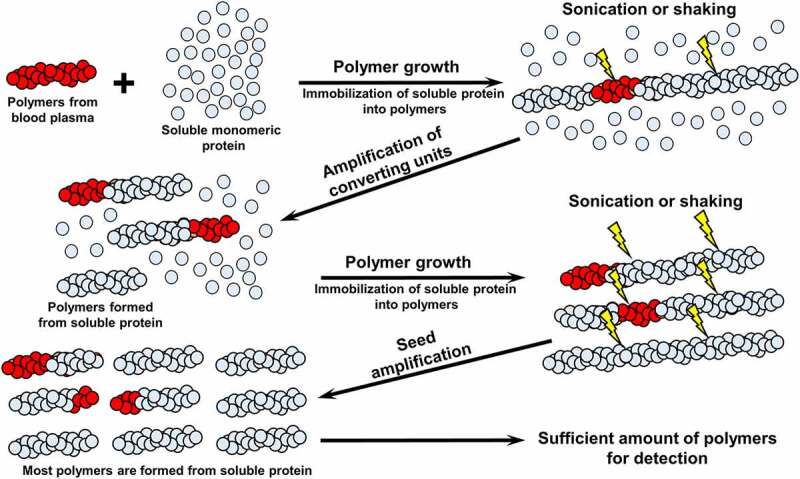
**The principle of protein misfolding amplification techniques (PMCA and RT-QuIC)**. Biological sample, potentially containing protein polymers is added to the excess soluble monomeric protein. During incubation, polymers are growing by incorporating monomers. Repetitive rounds of sonication (in case of PMCA) or vigorous shaking (in case of RT-QuIC) are applied in order to fragment fibrils into oligomeric seeds. After several cycles of amplification, sufficient amount of polymeric protein is produced for detection by ELISA, polymer fractionation followed by immunoblotting, or by binding to amyloid-specific dyes such as ThT

Initially, such a strategy has been successfully applied to the detection of the pathogenic ‘seeds’ of prion protein PrP, associated with Transmissible Spongiform Encephslopathies (TSEs, or prion diseases) in biological samples using an approach called **P**rotein **M**isfolding **C**ycling **A**mplification – **PMCA** [62,63] (Figure 4). In PMCA, a sample, containing small amounts of pre-existing polymers in amyloid conformation, that could be undetectable by standard biochemical protocols, is added to the solution of a monomeric protein with the same amino acid sequence. During incubation, polymers presented in the sample serve as nuclei or seeds for the polymerization of soluble protein. Repetitive cycles of amplification (usually interspaced with ultrasonic treatments, promoting fragmentation of the newly formed fibrils into new oligomeric seeds) produced sufficient amounts of fibrous polymers for the detection by standard assays such as ELISA, fluorescence microscopy, or aggregate sedimentation followed by immunoblotting. Number of cycles necessary for producing detectable amounts of polymeric material depends on the amount of polymers in the initial sample.

Later, a modified protocol named **Qu**arking **I**nduced **C**onversion (QuIC) was introduced [64], where automated tube shaking rather than sonication was used to fragment the aggregates, making the assay itself simpler and faster than original PMCA. In real-time modification of QuIC (RT-QuIC), PrP aggregation during the conversion is monitored by binding the fluorescent dye Thioflavin T (ThT), added to the reaction mix [65]. ThT binds to cross-β structures, thus detecting oligomers and fibrils that are in the amyloid conformation [66]. PMCA and RT-QuIC protocols are routinely employed for detection of the PrP-based prion in the meat and body liquids of domestic animals, as well as in natural environments [67]. RT-QuIC protocol for the detection of PrP aggregates in CSF has also been implemented into clinical practice for diagnostics of sporadic CJD in humans by The National CJD Research & Surveillance Unit (NCJRSU) in UK, and demonstrated 92% sensitivity and 100% specificity [68]. Amplification techniques were also applied to the detection of PrP aggregates in blood, urine and nasal swabs [69–71]. In 2018, US Centers for Disease Control and Prevention (CDC) included PrP RT-QuIC test in CSF or other tissues into the list of diagnostic criteria for probable CJD (https://www.cdc.gov/prions/cjd/diagnostic-criteria.html).

Successes with diagnostics of PrP-related pathologies using PMCA and QuIC techniques provide an incentive for the implementation of such approaches for the detection of polymeric forms of other amyloidogenic proteins in biological fluids. Recently, RT-QuIC has been applied to the detection of α-synuclein aggregation in brains and CSF from patients having dementia with Lewy bodies or Parkinson’s disease [72]. Several attempts were made to detect AβO in CSF and blood plasma by using various amplification protocols. Salvadores et al. [73] seeded polymerization of the soluble Aβ42 monomers by CSF samples, originated from patients with AD or non-AD neurodegenerative disorders, or with non-degenerative neurological diseases. Authors termed their amplification protocol Aβ-PMCA, although it employed shaking and ThT binding, similar to RT-QuIC. By comparing CSF samples to various amounts of synthetic Aβ42 seeds, authors concluded that Aβ-PMCA allows to detect as little as 3 fmol of AβO, and demonstrated an excellent performance of this assay in discrimination of AD patients from individuals suffering from other neurodegenerative or non-degenerative neurological disorders, with 90% sensitivity and 92% specificity. However, authors pointed to the quality of CSF samples as a limitation factor for the successful performance of Aβ-PMCA analysis [73].

Estrada et al. [74] applied PMCA to investigate the influence of imatinib, an inhibitor of a c-Abl kinase (enzyme, involved in the processing of Aβ protein precursor), on the levels of AβOs in blood plasma of transgenic AD mice. They have shown that imatinib reduces AβO levels in plasma, in an agreement with its ability to antagonize other AD-related features, such as accumulation of amyloid plaques in brain, neuroinflamation and cognitive deficits. AβO levels have been additionally monitored by ELISA, using amyloid conformation-specific antibodies 4G8, and results of immunoassay correlated with PMCA data. Thus, authors have shown potential applicability of the misfolded protein amplification technique to the analysis of AβO levels in blood, and correlation of this parameter with other AD markers.

**Figure 5.**
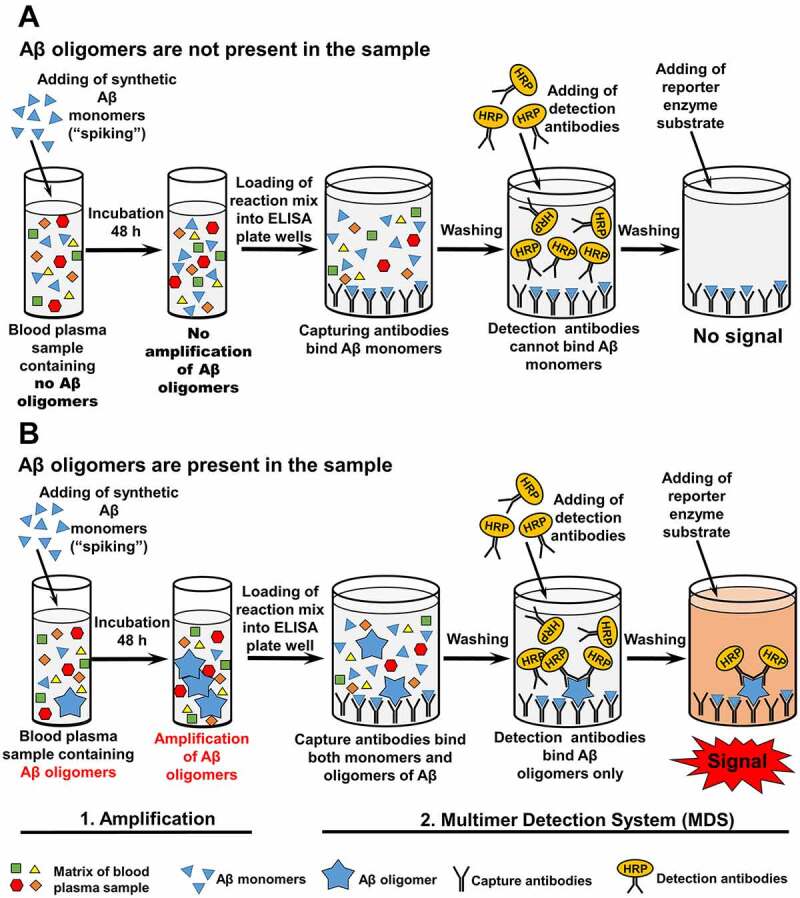
**Detection of Aβ oligomers in blood plasma**. This protocol consists of two sequential steps: 1) amplification of Aβ oligomers by ‘spiking’ of synthetic Aβ42 monomers into a blood plasma sample, followed by incubation for 48 h; 2) application of the Multimer Detection System [75], an approach allowing to eliminate the signal from monomeric Aβ, thus detecting only Aβ oligomers. For this purpose, different capturing and detection antibodies recognizing overlapped epitopes of Aβ are used. **A –** Aβ monomers are captured by an antibody attached to the surface of the ELISA plate. Detection antibody cannot bind Aβ monomers, because the single epitope is already occupied by capturing antibodies. No signal can be produced and detected when HRP substrate is added. **B** – Both Aβ monomers and oligomers are captured by an antibody attached to the surface of the ELISA plate. Unlike monomers, oligomers can bind detection antibodies because they possess numerous epitopes, of which some are not occupied by the capturing antibody. Thus the signal can be produced and detected when HRP substrate is added

A series of papers published recently by research consortium from Korea [75–77] represents the most advanced version of the prototype protocol for the detection of AβOs as a potential AD biomarker in blood using misfolded protein amplification techniques. To estimate AβO levels in blood plasma, soluble Aβ42 monomers were added (‘spiked’) into the aliquot of biological fluid, followed by the incubation of the mixture for 144 h. It has been shown that spiking of Aβ monomers to blood plasma samples led to an increase in AβO concentration, as measured by the increase of ThT fluorescence, when plasma sample originated from AD patient rather than normal control subject [76]. Incubation of the amplification reaction mixture was followed by detection of oligomers via the Multimer Detection System (MDS), an ELISA-based analytical platform that can specifically recognize multimeric form of an aggregated protein in the presence of soluble monomers (Figure 5 and [75] and references therein). To discriminate between AβOs and monomeric Aβ, epitope-overlapping antibodies specific to the N-terminus of Aβ were used in MDS for capturing and detecting AβOs. While Aβ monomers can also be captured, they lose the ability to bind a detection antibody, as the epitope is already occupied, and thus are eliminated from the further analysis. In contrast, captured AβOs retain the ability to bind detection antibodies, as they possessed multiple binding sites. AβO levels measured by amplification-MDS protocol were higher in patients with AD compared to control individuals, and correlated well with conventional AD biomarkers, such as Aβ42, P-tau and T-tau levels in CSF and PiB-SUVR imaging value [75].

Recent work by Youn et al. [77] reported further improvement of the AβO amplification technique. The analysis was conducted using commercially available kit (inBlood™ OAβ test), produced by People Bio Inc, Korea. Development of such a kit represents an important step towards the implementation of this protocol into every-day laboratory practice. Incubation time was reduced to 48 h. Blood plasma OAβ analysis using inBlood™ OAβ test kit allowed to discriminate AD patients from cognitively normal individuals with 100% sensitivity and 92% specificity. It is necessary to point out that researchers used a restricted number of samples, rarely exceeding 50 in each experimental or control group at each stage of protocol development thus their results need to be further validated clinically. Nevertheless, AβOs are promising candidates for highly sensitive and specific blood-based biomarker of AD that are available for sampling by a non-invasive procedure, and employ a relatively inexpensive protocol, with the equipment commonly available in most clinical labs. The crucial points for now are (a) validation of this approach at clinical level and (b) its optimization for early pre-symptomatic diagnostics.

## Amyloid-β oligomers in plasma: a promising candidate to «ideal» diagnostic marker for AD?

In 1998, The Ronald and Nancy Reagan Research Institute of the Alzheimer’s Association and the National Institute on Ageing Working Group have formulated the criteria for an “ideal” diagnostic marker for AD [78]. An “ideal” biomarker should be (1) able to detect a fundamental feature of AD neuropathology, (2) validated in neuropathologically confirmed AD cases, (3) precise (able to detect AD early in its course and distinguish it from other dementias), (4) reliable, (5) non-invasive, (6) simple to perform, (7) inexpensive. Appearance of polymeric forms of Aβ in CSF and blood is a unique feature of AD, and recent data reviewed above [73,77] indicate that analysis of AβO levels possesses a great potential for discriminating between AD and other types of neurodegenerative and neurological disorders with sufficient sensitivity and specificity. Use of blood plasma rather than CSF opens a possibility for non-invasive sampling of biological material. Application of PMCA or RT-QuIC-based techniques gives a chance to avoid use of complex protocols and expensive equipment and thus make the analysis simple to perform and inexpensive. To sum up, development of the clinically applicable protocol for the detection of AβO in blood plasma using PMCA, RT-QuIC or similar protein misfolding amplification technique can move us in the direction of establishing an “ideal” diagnostic biomarker for AD, potentially suitable for catching a disease at early presymptomatic stage.

## Future perspectives

**Figure 6.**
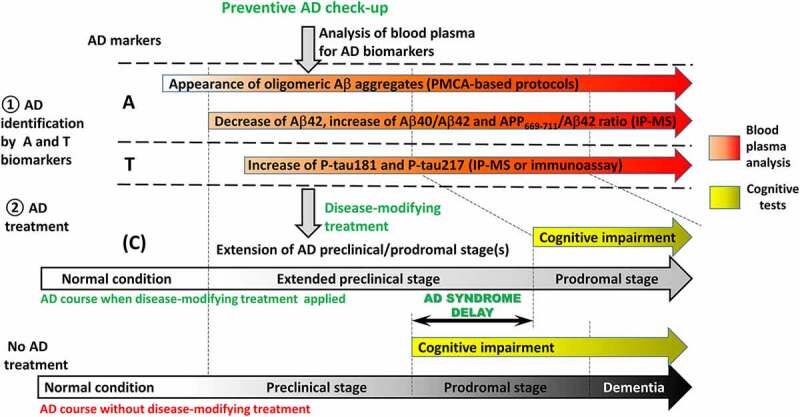
Prospective applications of blood plasma for early diagnostics of AD. Blood sampling is non-invasive procedure that is suitable for the implementation into preventive check-up for early identification of AD. Analysis of biomarkers specific for AD pathology (**A** and **T** blocks) gives a chance to identify AD at preclinical stage when patients are cognitively unimpaired. Application of disease-modifying treatment would allow to extend preclinical stage of disease and delay the development of AD syndrome and dementia

Availability of early preclinical diagnostics of AD is extremely important in a combination with approaches preventing disease propagation that are currently being developed although not yet proven in clinical trials. It is possible that failure of clinical trials held in the past is due to the fact that therapeutic approaches are effective only if applied early in disease progression [16]. To track drug performance and identify effects, one should have a reliable tool for a recurrent non-invasive, cheap screening that can distinguish alterations at the preclinical stage. An important step in this direction is the transition to the analysis of AD biomarkers in blood plasma. The non-invasive nature of blood sampling makes it possible to include AD biomarker analysis into prophylactic medical examination programme opening an opportunity for revealing AD at preclinical stage and giving a chance to apply potential disease-modifying therapy to delay clinical phase of disease (Figure 6). Nevertheless, Despite recent advances in identification of AD biomarkers in blood, expensive equipment, consumables and highly qualified staff are still necessary today to conduct the analysis like IP-MS. Non-invasive methods employing blood or other easily accessible tissue samples, performed in standard lab settings and capable of amplifying a weak signal, such as the QuIC or PMCA-based AβO detection, are the most promising for this purpose. Such techniques in application to protein misfolding diseases have a potential comparable to the use of PCR for diagnostics of viral infections. Recent developments in this direction provide a hope for establishing a relatively simple pre-symptomatic diagnostic assay in near future.
